# Age and Smoking Related Changes in Metal Ion Levels in Human Lens: Implications for Cataract Formation

**DOI:** 10.1371/journal.pone.0147576

**Published:** 2016-01-21

**Authors:** Alex Langford-Smith, Viranga Tilakaratna, Paul R. Lythgoe, Simon J. Clark, Paul N. Bishop, Anthony J. Day

**Affiliations:** 1 Wellcome Trust Centre for Cell-Matrix Research, Faculty of Life Sciences, University of Manchester, Manchester, United Kingdom; 2 School of Earth, Atmospheric and Environmental Sciences and Williamson Research Centre for Molecular Environmental Science, University of Manchester, Manchester, United Kingdom; 3 Centre for Ophthalmology and Vision Sciences, Institute of Human Development, University of Manchester, Manchester, United Kingdom; 4 Centre for Advanced Discovery and Experimental Therapeutics, University of Manchester and Central Manchester University Hospitals NHS Foundation Trust, Manchester Academic Health Science Centre, Manchester, United Kingdom; 5 Manchester Royal Eye Hospital, Central Manchester University Hospitals NHS Foundation Trust, Manchester, United Kingdom; Rush University Medical Center, UNITED STATES

## Abstract

Age-related cataract formation is the primary cause of blindness worldwide and although treatable by surgical removal of the lens the majority of sufferers have neither the finances nor access to the medical facilities required. Therefore, a better understanding of the pathogenesis of cataract may identify new therapeutic targets to prevent or slow its progression. Cataract incidence is strongly correlated with age and cigarette smoking, factors that are often associated with accumulation of metal ions in other tissues. Therefore this study evaluated the age-related changes in 14 metal ions in 32 post mortem human lenses without known cataract from donors of 11 to 82 years of age by inductively coupled plasma mass spectrometry; smoking-related changes in 10 smokers verses 14 non-smokers were also analysed. A significant age-related increase in selenium and decrease in copper ions was observed for the first time in the lens tissue, where cadmium ion levels were also increased as has been seen previously. Aluminium and vanadium ions were found to be increased in smokers compared to non-smokers (an analysis that has only been carried out before in lenses with cataract). These changes in metal ions, i.e. that occur as a consequence of normal ageing and of smoking, could contribute to cataract formation via induction of oxidative stress pathways, modulation of extracellular matrix structure/function and cellular toxicity. Thus, this study has identified novel changes in metal ions in human lens that could potentially drive the pathology of cataract formation.

## Introduction

Age-related cataract is the cause of blindness in 51% of the world’s 39 million blind people [1]. Although treatable by the removal of the lens and the insertion of an artificial intraocular prosthesis, this surgical approach is not possible for the majority of blind people in the developing word due to cost and access to medical facilities [2]. Therefore further research is required to understand the cause of cataract formation to identify novel ways of slowing progression and delaying the need for surgery.

Cataract incidence is strongly associated with ageing worldwide [3–5]; for example in Australia it affects ~2.6% of women and ~3.7% of men between the ages of 40 and 50, and the incidence approximately doubles with every subsequent decade of life, until all are affected over the age of 90 [3]. Other risk factors for cataract include gender [6] (women are at greater risk), diabetes [6] and infrared [7] and UV-B [2] radiation. Being a current smoker strongly increases the risk of cataract (Odds ratio (OR) 1.45) and stopping smoking reduces your risk (OR 1.31) but it remains increased compared to those who have never smoked [6, 8].

Changes in metal ion levels occur as a consequence of normal ageing in many tissues and this is often influenced by smoking status [9–18]. As outlined in Table 1, there has been one previous study that has determined the age-related change in metal ions in clear (i.e. non-cataract) human lenses and only 3 metal ions were analysed; there was a significant age-related increase in the amount of cadmium (Cd), a trend towards a decrease in copper (Cu) and no detectable lead (Pb). Furthermore, tobacco smoke and even electronic cigarette smoke is known to contain a large number of different metal ions [19, 20] but no study has examined the effect of smoking on their accumulation in lenses without known cataract. The levels of metal ions in human lenses with cataract (i.e. removed during cataract surgery) has been more extensively investigated [18, 21–34] (see Table 1), but these have not distinguished between disease-related and age-related changes.

**Table 1:** Existing literature on metal ions in human lens.

Publication	Rajkumar 2013 [21]		Erie 2005 [18]	Cekic 1998 and 1999 [22–24]				Hou 1996 [25]		Shukla 1996 [26]		Karakucuk 1995 [27]		Srivastava 1992 [28]	
Population^#^	Non-cataract 5, Cataract 28		Non-cataract 16 (10m + 6f)	Non-cataract 9 (5m + 4f), Cataract 37 (25m + 12f)				Non-cataract 36, Cataract 36		Non-cataract10, Cataract 50		Non-cataract 9, Cataract 36		Not reported	
Location	India		United States	Turkey				China		India		Turkey		India	
Method^†^	AAS		ICP-MS	AAS				GRS		AAS		AAS		AAS	
	μg/g dry weight^§^	Cataract Change^‡^	μg/g dry weight	μg/g dry weight^§^	Age Change (38–59 vs ≥60)^‡^	Cataract Change^‡^	Gender Difference^‡^	μg/g dry weight^§^	Cataract Change^‡^	μg/g dry weight^§^	Cataract Change^‡^	μg/g dry weight^§^	Cataract Change^‡^	μg/g dry weight^§^	Cataract Change^‡^
Al*				1.152 ± 0.598		1.6↓	No								
Sb*								0.038 ± 0.026	2.2↑						
Ar*															
Cd*			0.013 ± 0.018	0.045 ± 0.004	2.5↑	22↑	↑m								
Cs*								0.060 ± 0.012	1.5↓						
Ce*								0.203 ± 0.122	1.5↑						
Cr*				1.97 ± 1.007		2.5↓	No	0.53 ± 0.19	1.8↓						
Co*								0.040 ± 0.018	1.4↓						
Cu*	3.78 ± 1.33	~2↑ns		0.69 ± 0.15	1.2↓ns	3.1↑	No			0.4 ± 1.2	No			0.25 ± 0.06	10.9↑
Fe*				17.340 ± 7.146		1.7↑	No	35.3 ± 6.3	1.7↑						
Pb*			0.020 ± 0.018	0	n/a	↑	No			3.0 ± 1.2	37↑				
Mg*								210 ± 38	No						
Mn*	1.62 ± 0.37	~2↑		1.397 ± 0.931		3↓	No	1.16 ± 0.64	4.1↓						
Hg*			0												
Mo*															
Ni*				0.422 ± 0.253		5.4↑	No								
K*								6720 ± 1280	5.3↓	8500 ± 320	4.5↓				
Rb*								7.22 ± 1.42	5.4↓						
Sc*								0.019 ± 0.005	1.2↓						
Se*								0.540 ± 0.187	No			4.43 ± 2.53	No		
Na*								1320 ± 740	6.8↑	2900 ± 2300	No				
Tl*			0					0.014 ± 0.004	No						
V*															
Zn*	33.45 ± 3.41	~2.5↑						25.7 ± 4.4	No	280 ± 82.2	1.7↓			12.8 ± 2.6	1.8↑

# The number of samples in the study and gender (male (m) or female (f)), where reported, are included

† Method of analysis: atomic absorption spectroscopy (AAS), inductively coupled plasma mass spectrometry (ICP-MS) and gamma-ray spectroscopy (GRS).

*Indicates that this metal ion was quantified in the present study.

§ Data are presented as the mean ± standard deviation

‡ A significant increase in age, gender or cataract is indicated by the fold increase and an up arrow (↑), a decrease by a fold decrease and down arrow (↓) and no change by “No”. Where there is a clear trend that does not reach significance the abbreviation ns for not significant is included. The abbreviation n/a indicates not applicable and ~ indicated that the fold change was estimated from the data presented. For lead the fold increase with cataract in Cekic 1998 and 1999 could not be calculated as no lead was detectable in normal lens.

Thus, in this study we determined the levels of 14 metals (i.e. aluminium (Al), arsenic (As), Cd, chromium (Cr), cobalt (Co), Cu, iron (Fe), Pb, manganese (Mn), molybdenum (Mo), nickel (Ni), selenium (Se), vanadium (V) and zinc (Zn)) in lens tissue from 32 human donors without known cataract, for which the smoking status was documented for 24 of these. This has identified novel changes in metal ions that might play a role in cataract initiation and progression.

## Methods

### Sample collection

Post mortem human eyes were obtained from the Manchester Royal Eye Hospital Eye Bank after removal of the corneas for transplantation. None of the donors had been diagnosed with cataract and there was no reporting of cataract by their next of kin (such reporting is a requirement before use of corneas for transplant); moreover we only used lenses without obvious opacity in our study. That notwithstanding we cannot rule out that some degree of cataract may have been present [3–5] (i.e. molecular changes consistent with early stage disease), but it was not possible for us to assess this post mortem; diagnosis of the presence or absence of early cataract requires a detailed slit lamp examination including retro-illuminated observations [35]. Furthermore, none of the donors had any other diagnosed/reported form of eye disease. In all cases prior written, informed consent had been obtained for the ocular tissue to be used for research, and guidelines established in the UK Human Tissue Act 2004 were followed. Ethical approval was given by the University of Manchester Ethics Committee No. 3 (reference number 11305). Our research adhered to the tenets of the Declaration of Helsinki. Thirty-two lenses from 32 different human donors (See S1 Table) were removed from eye tissue using titanium instruments (Ti Alloy 6AL 4V, ASTM B348 GRADE 5) and nitric acid- (trace metal grade, VWR International) cleaned plastic wear to avoid contamination [36]. Lenses were stored at -80°C in trace metal analysis vacutainer tubes (Becton Dickinson). Donors ranged from 11 to 82 years and comprised 11 females and 21 males. Based on next of kin reporting 10 donors were smokers and 14 were non-smokers; the smoking status of the other donors was not known.

### Sample processing for ICP-MS metal analysis

The intact lenses were heated at 50°C for ~2 days to dry the tissue prior to weighing (to within ±0.1 mg). The samples were then processed as previously described [36]. Briefly, the lens tissue was dissolved in trace metal grade concentrated (67% v/v) nitric acid (820 μl), 30% (v/v) hydrogen peroxide (180 μl) (Sigma-Aldrich) and high purity deionised water (100 μl; 18.2 MΩ) and microwave digested (MARSXpress, CEM Corporation) in a closed system (100% power 400W, 5 min ramp to 75°C, 30 min incubation, 5 min ramp to 95°C and 60 min incubation). Prior to analysis by inductively coupled plasma mass spectrometry (ICP-MS, Agilent 7500cx) the samples were diluted to 4 ml with high purity deionised water; ~2.5 ml of each sample was then analysed essentially as described previously [36]. Background levels of metal ions were determined from the mean average measurement of four samples processed as above but containing no tissue [36]. These were deducted from the experimental values for the lens samples followed by normalisation of all metal ion levels on the basis of the dry weight of the tissue.

### Statistical analysis

The correlation between age and metal ions, and between different metal ions, was determined using the Pearson correlation with Student’s t-distribution to calculate significance. Differences in metal ions between age groups were determined by One-Way ANOVA with Tukey *post hoc* analysis in JMP 11. Student’s t-test was used to determine the significance of differences in metal ions between gender and between smoking status.

## Results

### Metal ion composition of human lens

**Fig 1:**
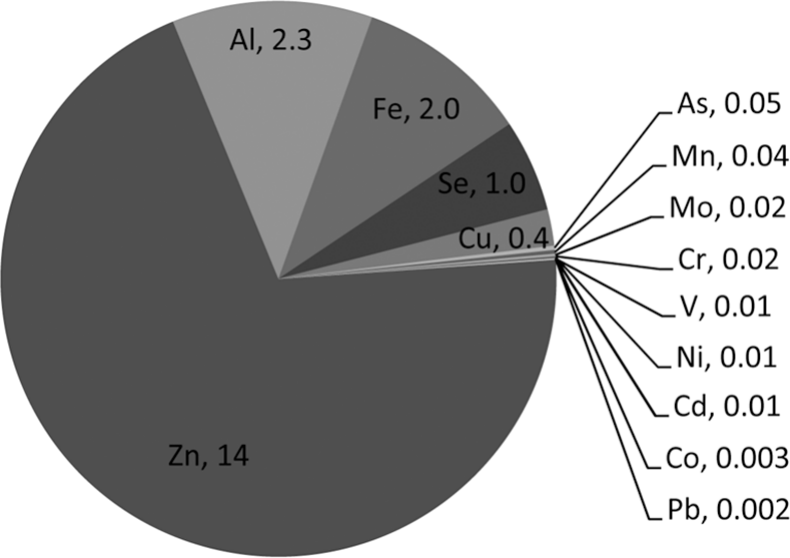
Comparison of metal ion levels in human lens. Pie chart showing the relative proportion of the 14 metal ions quantified in human lens tissue without known/obvious cataract. The mean average value (in μg/g dry weight tissue) is shown next to the atomic symbol.

**Fig 2:**
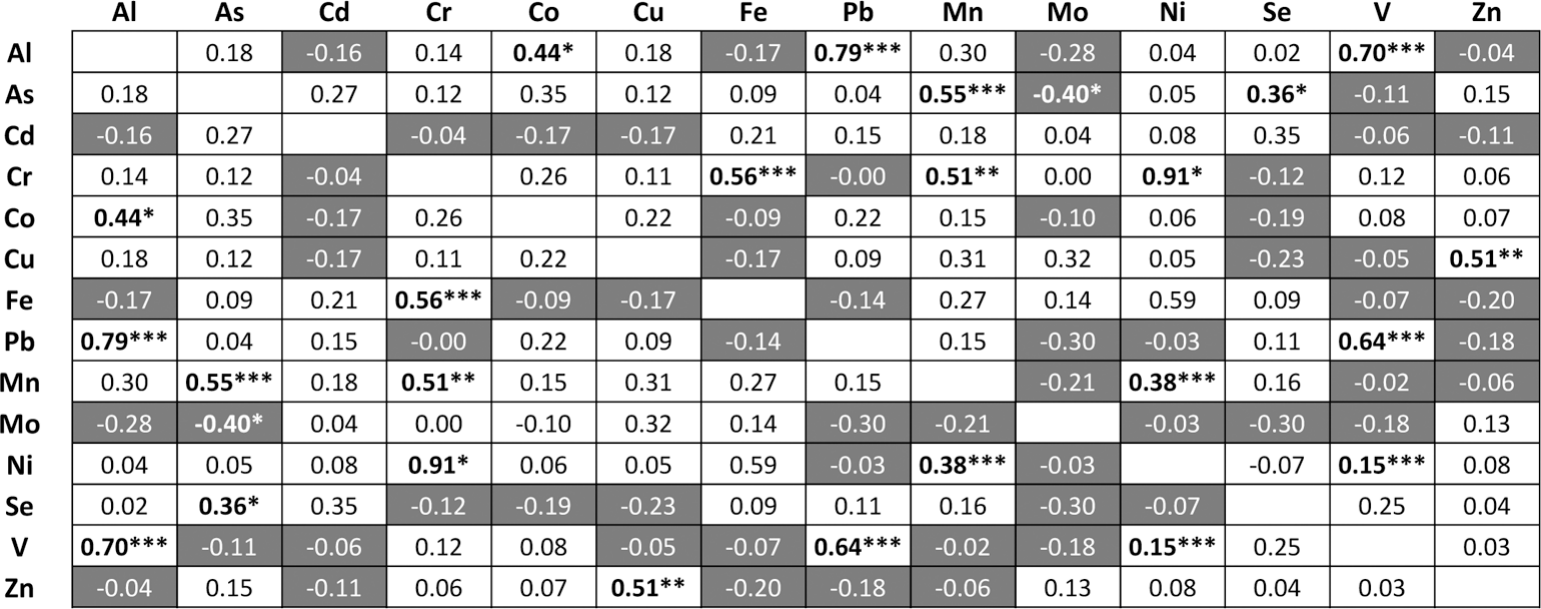
Pearson correlation between metal ions. Numbers on a white or grey background show a positive or negative Pearson correlation respectively. Significant correlations were determined by Student’s t-distribution and denoted by * = p<0.05, ** = p<0.01 and *** = p<0.001.

The amounts of 14 metal ions (i.e., Al, As, Cd, Cr, Co, Cu, Fe, Pb, Mn, Mo, Ni, Se, V and Zn) were determined in the lenses of 32 human donor eyes (without known/obvious cataract) after background subtraction and normalisation to dry weight of tissue; it should be noted that we used entire lenses in our analysis so that the data obtained was comparable with the studies in Table 1. Fig 1 and Table 2 show the mean values from all of the samples, where Zn was the most abundant metal ion (13.6 μg/g), followed by Al (2.3 μg/g), Fe (2.0 μg/g), Se (1.0 μg/g) and Cu (0.41 μg/g) with other metal ions being present at levels less than 0.1 μg/g of dry weight of tissue. When the individual metal ions were correlated with each other on a donor by donor basis (Fig 2) a number of correlations were identified; e.g. Al positively correlated with the amount of Co, Pb and V (p<0.05, p<0.001 and p<0.001 respectively), Cu and Zn were positively correlated with each other (p<0.01), Fe positively correlated with Cr (p<0.001) and Se positively correlated with As (p<0.05).

**Table 2:** Levels of metal ions in human lens without known cataract.

	Al	As	Cd	Cr	Co	Cu	Fe	Pb	Mn	Mo	Ni	Se	V	Zn
**Mean** μ**g/g dry weight**	2.3	0.050	0.0058	0.017	0.0029	0.41	2.0	0.0018	0.044	0.018	0.010	1.0	0.0058	13.6
**Standard deviation**	2.1	0.024	0.0082	0.027	0.0014	0.16	1.8	0.0017	0.016	0.020	0.012	0.3	0.0074	2.2
**Minimum**	0.0	0.000	0.0000	0.002	0.0008	0.16	0.4	0.0000	0.005	0.004	0.002	0.5	0.0008	10.3
**Maximum**	10.4	0.093	0.0404	0.157	0.0088	0.79	7.1	0.0083	0.089	0.093	0.070	1.9	0.0376	18.4
**% Coefficient of variance**	94	48	141	163	50	39	89	92	37	110	126	30	127	16

### Age-related changes in metal ions

**Fig 3:**
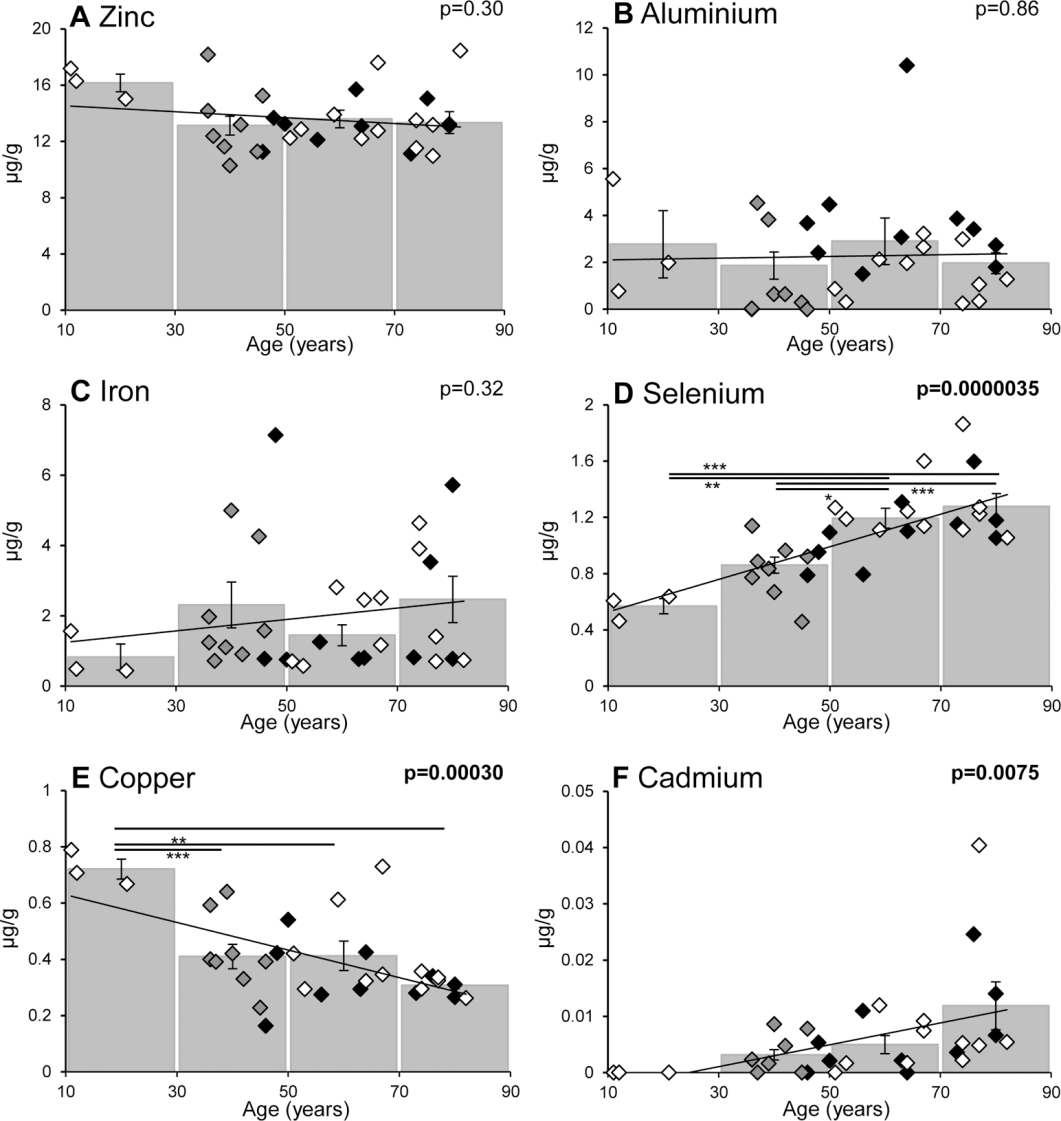
Age-related changes in metal ions in human lens. The age-related change in 6 out of the 14 metal ions analysed in lenses from human donor eyes, including the 5 most abundant metal ions (A) Zinc, (B) Aluminium, (C) Iron, (D) Selenium and (E) Copper, and also (F) Cadmium. Metal ion levels for individual donor samples (n = 32), and the mean values (± S.E.M) for multiple donor tissues between 11–30, 31–50, 51–70, and 71–90 years, are plotted against age. Black diamonds denote smokers, white diamonds denote non-smokers and grey diamonds show donors of unknown smoking status. P values represent the significance of the Pearson correlation between age and metal ion as determined by Student’s t-distribution. Significant differences between age ranges was calculated by one-way ANOVA with Tukey *post hoc* analysis; horizontal lines indicates which pairs of values are significant (* = p<0.05, ** = p<0.01 and *** = p<0.001).

**Fig 4:**
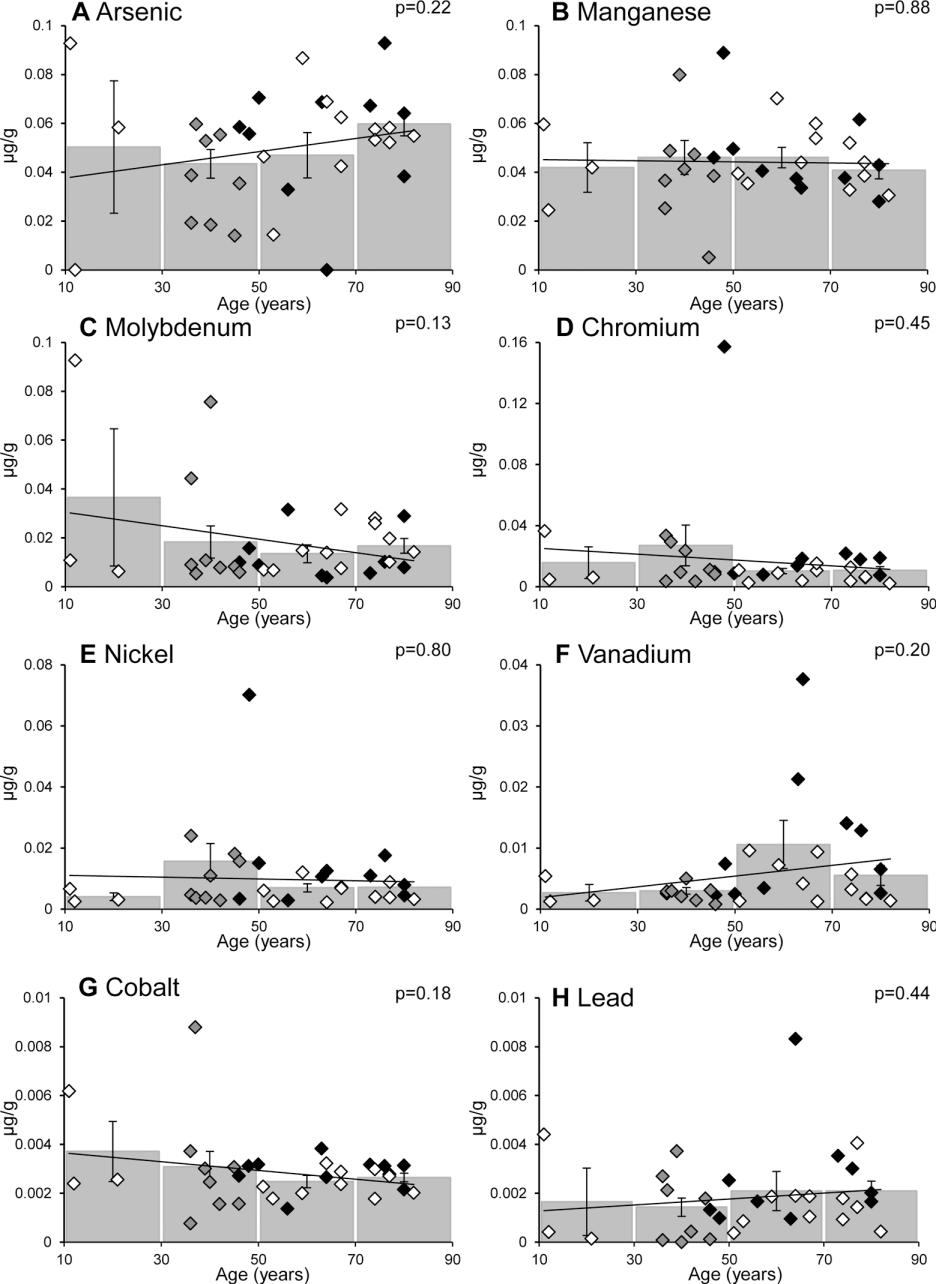
Age-related changes in metal ions in human lens. The age-related change in 8 out of 14 metal ions in human lens tissues. (A) Arsenic, (B) Manganese, (C) Molybdenum, (D) Chromium, (E) Nickel, (F) Vanadium, (G) Cobalt and (H) Lead. Metal ion levels for individual donor samples (n = 32), and the mean values (± S.E.M) for multiple donor tissues between 11–30, 31–50, 51–70, and 71–90 years, are plotted against age. Black diamonds denote smokers, white diamonds denote non-smokers and grey diamonds show donors of unknown smoking status; p values represent the significance of the Pearson correlation between age and metal ion as determined by Student’s t-distribution. There were no significant differences between the age ranges analysed.

When the 14 metal ions were analysed against donor age a number of correlations were observed (see Fig 3 and Fig 4). Of the 5 most abundant metal ions (Zn, Al, Fe, Se and Cu) only Cu and Se changed significantly, with the former decreasing (Fig 3E) and the later increasing (Fig 3D) with age; when non-smokers alone were analysed age-related changes were still apparent (with p = 0.0014 and p = 0.0011 for Cu and Se, respectively). In the case of Cu, this metal ion decreases most dramatically over the first 50 years (p = 0.0029 for a comparison of 11–30 year donors with those 31–50 years of age) with no significant differences between the 31–50, 51–70 and 71–90 age groups (Fig 3E). In contrast, Se increases more linearly with age (i.e. in a progressive manner) with significant differences between most of the age groups (Fig 3D). Of the 9 less abundant metal ions (i.e. As, Mn, Mo, Cr, Ni, Cd, V, Co, Pb) only Cd shows a significant correlation with age (Fig 3F and Fig 4).

### Smoking and gender related differences in metal ions

**Fig 5:**
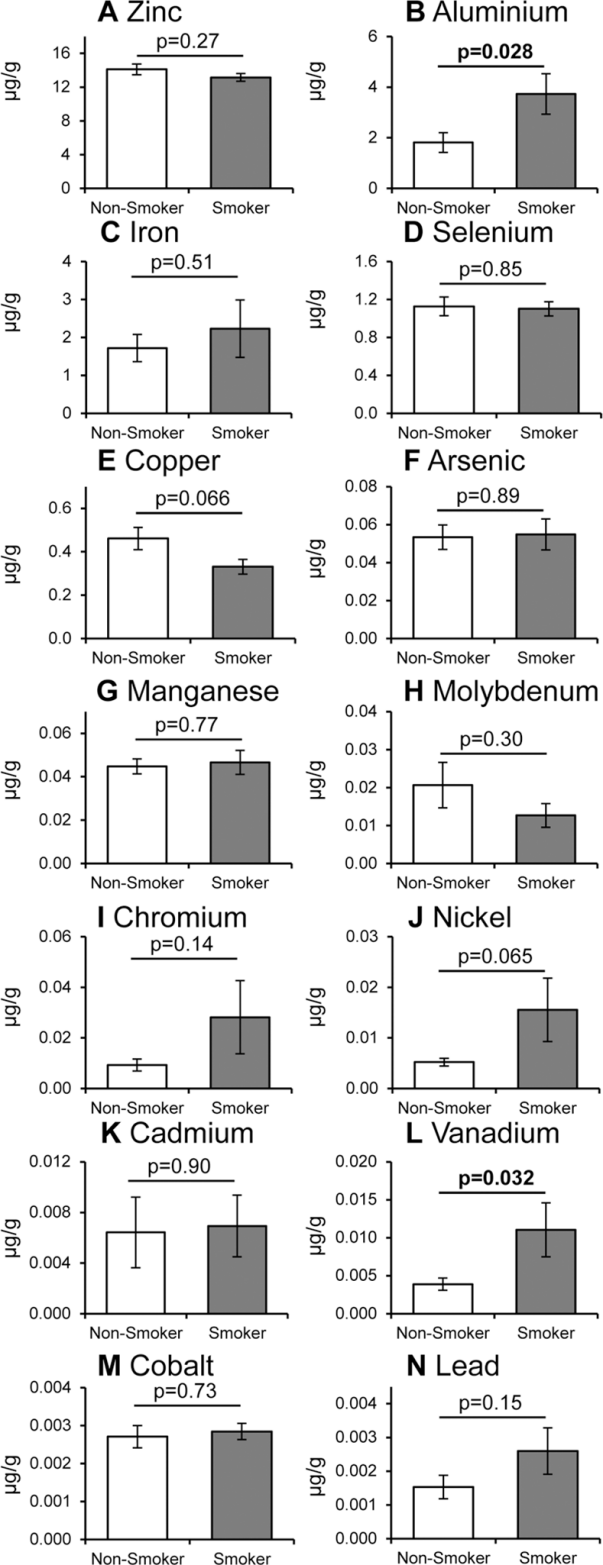
Smoking-related changes in metal ions in human lens. Comparison of the mean values (±S.E.M.) of 14 metal ions in human lens tissue from smokers (n = 10) and non-smokers (n = 14): (A) Zinc, (B) Aluminium, (C) Iron, (D) Selenium, (E) Copper, (F) Arsenic, (G) Manganese, (H) Molybdenum, (I) Chromium, (J) Nickel, (K) Cadmium, (L) Vanadium, (M) Cobalt and (N) Lead ions; p values determined by Student’s t-test are shown. There was no significant difference in the ages of the smokers vs. non-smokers in this analysis.

**Fig 6:**
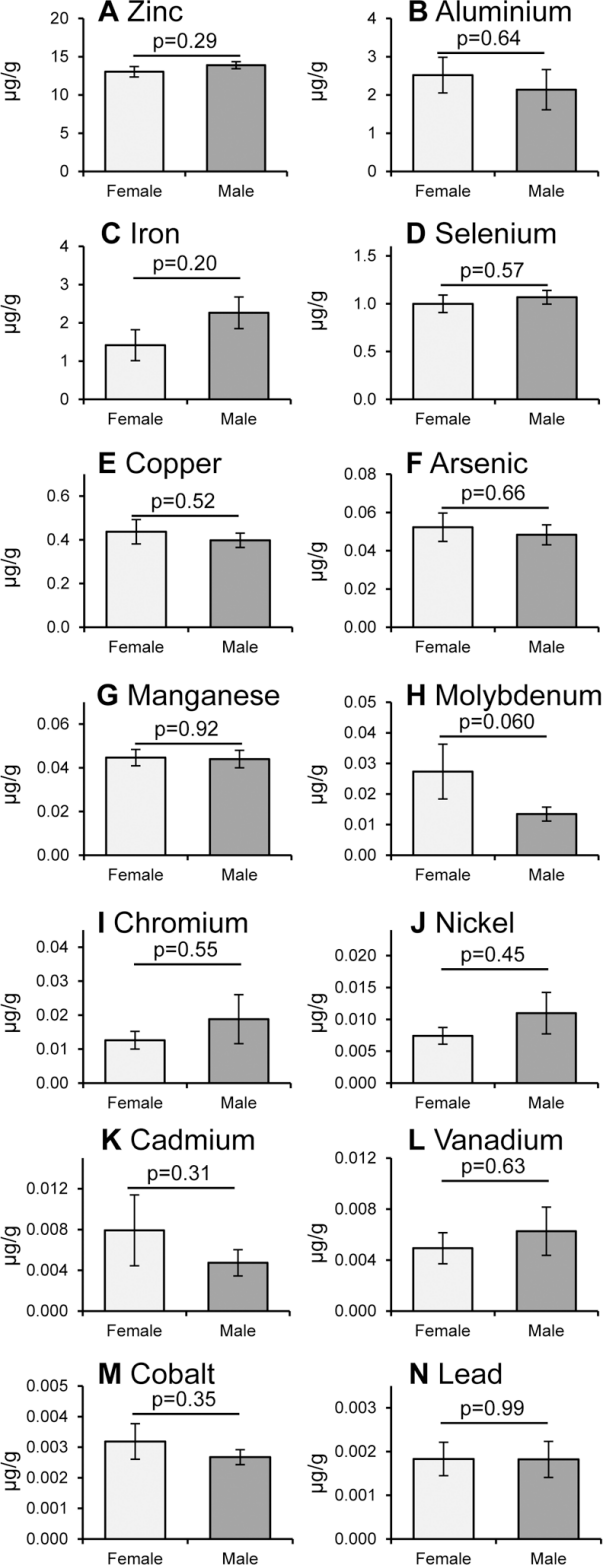
Gender-related differences in metal ions in human lens. Comparison of the mean values (±S.E.M.) of 14 metal ions in human lens tissue from females (n = 11) and males (n = 21): (A) Zinc, (B) Aluminium, (C) Iron, (D) Selenium, (E) Copper, (F) Arsenic, (G) Manganese, (H) Molybdenum, (I) Chromium, (J) Nickel, (K) Cadmium, (L) Vanadium, (M) Cobalt and (N) Lead ions; p values determined by Student’s t-test are shown. There was no significant difference in the ages of the males and females donors analysed here.

As discussed in the introduction, smoking is a major risk factor for cataract [6, 8] and metal ions are known to be present in cigarette smoke [19, 20]. Therefore, we compared the levels of metal ions in the lens samples from donors who were smokers (10) or non-smokers (14). The amount of Al (Fig 5B) and V (Fig 5L) were significantly increased in smokers (p = 0.028 and p = 0.032, respectively) and there was a clear trend for Ni, Cr and Pb (Fig 5J, 5I and 5N, respectively) that didn’t reach significance. There was also a trend towards a decrease in the amount of Cu (Fig 5E, p = 0.066). Women have an increased risk of cataract [6], however, no significant differences in metal ions were measurable with gender (Fig 6). However, there was a trend towards a decreased amount of Mo in men compared to women (Fig 6H, p = 0.060).

## Discussion

Here we have determined that there are age- and smoking-related changes in metal ion levels in the lenses from human eye donors without known or obvious cataract or indeed any other known eye pathology. This is the first study to quantify metal ions in lenses from smokers without known cataract and also represents the most comprehensive investigation of metal changes with age in that we determined the amounts of 14 metal ions in 32 donors across a wide age range (11–82). A potential limitation of this study is that we analysed the entire lens rather than dividing it into sub-regions given that changes could potentially be restricted to a particular part of the lens. However, it should be noted that all of the previous studies described in Table 1 also analysed the entire lens (i.e. allowing direct comparison with our data) and recent work has shown that there are no significant differences in the distribution of metal ions (Cu, Fe, Zn) between the capsule and the nuclear and cortical regions of human lens [37].

We investigated the effect of ageing on metal ion accumulation since age is a major risk factor for cataract formation [6, 8] and metal ions are known to have a wide range of biological/toxic effects in many different tissues [38–40]. Importantly, we observed a statistically significant age-related increase in the levels of Cd and Se and a significant decrease in Cu ions (see Fig 3). These findings are consistent with the study from Cekic *et al*. (1998), based on 9 non-cataract samples, that identified a significant increase in the amount of Cd and a trend towards a decrease in Cu with age (see Table 1) [23]; Watts (1994) also reported that Se levels increases with age, although no data or population details were presented [41]. In addition, Se has also been observed to preferentially accumulate in the lens of the zebrafish eye compared to other tissues [42]. Cd ions have previously been observed to increase with age in serum [9] and in other ocular tissues (retinal pigment epithelium (RPE) and choroid), although only in females [10]; however, we saw no gender-specific changes in Cd or any metal ions in our lens samples (Fig 6). As in our study, decreased levels of Cu have been observed with age in some parts of the brain [11] and a decrease in Cu has been associated with Alzheimer’s, Parkinson’s, age-related macular degeneration (AMD) and diabetes [43–49]; whereas, age-related increases in Cu have been seen in other ocular tissues (e.g. RPE or choroid) [10].

This is the first study to measure the levels of As, Mo and V in lens, where the average values for these and the other 11 metal ions are shown in Fig 1 and Table 2. The levels of Al, Cu, Se and Zn we determined are very similar to previous studies, however, we observed much lower levels of Cd, Cr, Co, Fe, Pb, Mn and Ni than seen previously (see Table 1). These observed differences could be due to the analysis technique used; only Erie *et al*. (2005) [18] used ICP-MS (as in our study), but provided values for just 2 out of the 14 metal ions that we included. ICP-MS is considered to be more sensitive than atomic absorption spectroscopy (AAS), which was used in most of the other studies, with a larger linear range for quantification and lower limit of detection [50]. Alternatively, the differences could be geographical and/or environmental. As shown in Table 1, none of the other studies analysed samples from the UK population. Environmental differences in Cd, Cr, Fe, Mg (that we did not analyse), Mn, Pb and Zn have previously been observed between individuals living in rural and urban environments within the same country [33, 34], however we did not have access to this donor information.

Smoking is also a major risk factor for cataract formation [6, 8] and is well established to be a major source of metal ions [19, 20]. However, there has been no comparison of metal ion levels in lens tissues from human donors without known cataract based on their smoking status. Here we observed a significant increase in Al and V ions in the lenses from smokers compared to non-smokers, a trend towards an increase in Cr, Ni and Pb and a trend towards a decrease in Cu ions (Fig 5). Increased Al levels have been observed in blood [51], saliva [12] and hair [13] of smokers; however, there was no significant differences in Al in erythrocytes, plasma, serum or urine [15, 17]. While V is known to be present in cigarettes [17], changes with smoking have not been documented elsewhere.

Statistically significant differences in metal ions have also been observed when comparing cataract vs non-cataract human lens tissues (see Table 1) [21–28], however, smoking status was not reported in any of these studies. For example, an increase in Fe and Pb and a decrease in Cr and K were seen consistently (that is where these metal ions were analysed), whereas we saw no significant age-related alterations in Fe, Pb or Cr, and K levels were not determined in our study. Cd ions were found to be greatly increased in cataract as well as increasing significantly with age (Fig 3), however, much higher levels were present in the disease-associated lens tissue [22, 23]. Al and Co decease with cataract [25], whereas Ni increases [24], but there were no age-related alterations in our study. On the other hand, we saw an age-related increase in Se where no difference was observed in cataract and an age-related decrease in Cu where in cataract Cu was increased or unchanged [21–23, 26, 28]. Thus, we hypothesise (based on a comparison of our data with those in the literature) that changes in Cd, Co, Cu, Fe, Ni and Pb seen in cataract are likely to be associated with cataract disease pathology, however, it is not clear whether any of these metal ions have a causative role and further studies are needed to test this posibility. Our findings that there are significant changes in Al, Cd, Cu, Se and V with age or smoking (Figs 2 and 3) implicate these metal ions in the initiation of cataract, whereas the disease-related changes described above might only occur during its progression; again, additional research is needed to investigate this further. Consistent with these suggestions, topical administration of a divalent metal ion chelator reduced lens clouding (and lipid peroxidation) in a rat model of cataractogenesis [52].

As noted above there is an age-related increase in Cd in human lens (see Fig 3), where this metal ion is known to be a potent inducer of oxidative stress [38], which is thought to be a major pathogenic pathway in cataract formation [53]; the smoking-related changes that we identified in Al and V could also cause oxidative stress and the production of reactive oxygen species (ROS) [54, 55]; e.g. Al catalyses the reduction of Fe^3+^ to Fe^2+^ and thus promotes the Fenton reaction [56]. We also observed an age-related increase in Se, which is a component of selenocysteine, a crucial amino acid in oxidative stress defence proteins [57–60]. Se deficiency is also reported to cause cataract [41], however, dietary supplementation with Se for an average of ~5.5 years did not decrease its incidence [61]. Conversely, Se injections are used to induce cataract formation in experimental models via the generation of reactive oxygen species [62–64]. Thus, in this study we cannot discriminate between an increase in Se in the lens with age due to an up-regulation of Se-containing antioxidant enzymes, which could be protective, or a toxic accumulation of Se that could cause oxidative stress. The age-related decrease in Cu (Fig 3) could also impair the function of antioxidant enzymes that require Cu ions as cofactors (e.g. superoxide dismutase 1) [21].

Aside from directly causing oxidative stress, metal ions (e.g. Cd, Co, Cu, Fe, Pb, Zn) [65] can also affect redox sensitive signalling pathways as well as other mechanisms of cellular toxicity [66, 67]. For example, Cd can induce ROS and modulate expression of various genes via the Nrf2 transcription factor [65]. In the lens the X_c_^-^ cystine/glutamate exchanger is an important part of ROS defence [68, 69] and its expression has been found to decrease in the human lens with age [70]. Given that the expression of X_c_^-^ can be controlled by the Nrf2 transcription factor [71], we speculate that this pathway could be affected by alterations in Cu, Cd, Al and V, e.g. with age and smoking.

Changes in metal ions could also affect extracellular matrix, which is an important component of lens tissue and is essential for transparency [72, 73]. For instance, heparan sulphate (HS) plays a vital role in lens matrix organisation such that reductions in this glycosaminoglycan (GAG), associated with mutations in the perlecan gene (*Hspg2*), result in cataract in man [74]; conversely, infrared irradiation, a risk factor for cataract [7], leads to an increase in lens HS [73]. This is potentially important since HS (and other related GAGs) sequester divalent cations and thus age/disease-related changes in these matrix molecules [75–77] are likely to affect the availability/activity of metal ions. Moreover, metal ions (e.g. Cd) can cause changes in the levels, composition and function of HS in other tissues [78–81] as well as affecting matrix integrity through ROS- and metal ion-mediated degradative processes [82–85]. Therefore, although the direct effects of metal ions on lens matrix have not been studied, it seems possible that this may contribute to the aetiology of cataract formation and disease progression.

## Conclusion

In the human lens we have observed age-related changes in Cd, Cu and Se ions and changes in Al and V with smoking. These alterations in metal ions could contribute to cataract formation by inducing oxidative stress, impairing anti-oxidant pathways or modifying the structure/function of the lens extracellular matrix. Importantly, the data we have described here provides a good basis for further mechanistic studies to determine whether this is indeed the case.

## Supporting Information

S1 Raw Data Details of donors, gender, smoking status and metal ion levels. (XLSX)

S1 Table Details of lens donors. (PPTX)
