# Supplementary material for: Age and Smoking Related Changes in Metal Ion Levels in Human Lens: Implications for Cataract Formation
Source: PLoS One. 2016 Jan 21;11(1):e0147576. doi: 10.1371/journal.pone.0147576 (PMC4721641; doi:10.1371/journal.pone.0147576)
Supplement: S1 Table — (PPTX) [file pone.0147576.s002.pptx]

## Slide 1
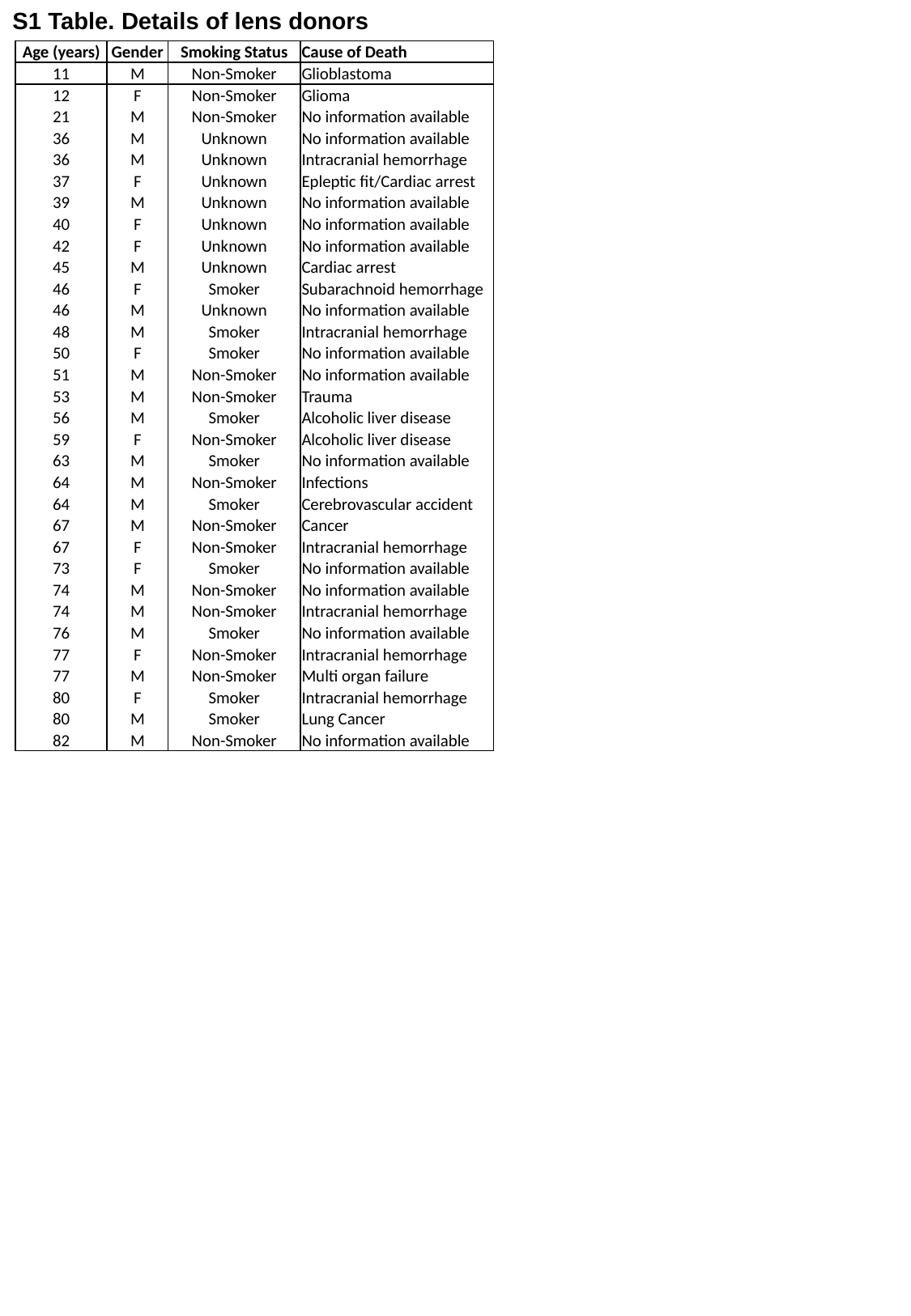

S1 Table. Details of lens donors
| Age (years) | Gender | Smoking Status | Cause of Death |
| --- | --- | --- | --- |
| 11 | M | Non-Smoker | Glioblastoma |
| 12 | F | Non-Smoker | Glioma |
| 21 | M | Non-Smoker | No information available |
| 36 | M | Unknown | No information available |
| 36 | M | Unknown | Intracranial hemorrhage |
| 37 | F | Unknown | Epleptic fit/Cardiac arrest |
| 39 | M | Unknown | No information available |
| 40 | F | Unknown | No information available |
| 42 | F | Unknown | No information available |
| 45 | M | Unknown | Cardiac arrest |
| 46 | F | Smoker | Subarachnoid hemorrhage |
| 46 | M | Unknown | No information available |
| 48 | M | Smoker | Intracranial hemorrhage |
| 50 | F | Smoker | No information available |
| 51 | M | Non-Smoker | No information available |
| 53 | M | Non-Smoker | Trauma |
| 56 | M | Smoker | Alcoholic liver disease |
| 59 | F | Non-Smoker | Alcoholic liver disease |
| 63 | M | Smoker | No information available |
| 64 | M | Non-Smoker | Infections |
| 64 | M | Smoker | Cerebrovascular accident |
| 67 | M | Non-Smoker | Cancer |
| 67 | F | Non-Smoker | Intracranial hemorrhage |
| 73 | F | Smoker | No information available |
| 74 | M | Non-Smoker | No information available |
| 74 | M | Non-Smoker | Intracranial hemorrhage |
| 76 | M | Smoker | No information available |
| 77 | F | Non-Smoker | Intracranial hemorrhage |
| 77 | M | Non-Smoker | Multi organ failure |
| 80 | F | Smoker | Intracranial hemorrhage |
| 80 | M | Smoker | Lung Cancer |
| 82 | M | Non-Smoker | No information available |
